# Bound2Learn: a machine learning approach for classification of DNA-bound proteins from single-molecule tracking experiments

**DOI:** 10.1093/nar/gkab186

**Published:** 2021-03-21

**Authors:** Nitin Kapadia, Ziad W El-Hajj, Rodrigo Reyes-Lamothe

**Affiliations:** Department of Biology, McGill University, 3649 Sir William Osler, Montreal, QC H3G 0B1 Canada; Department of Biology, McGill University, 3649 Sir William Osler, Montreal, QC H3G 0B1 Canada; Department of Biology, McGill University, 3649 Sir William Osler, Montreal, QC H3G 0B1 Canada

## Abstract

DNA-bound proteins are essential elements for the maintenance, regulation, and use of the genome. The time they spend bound to DNA provides useful information on their stability within protein complexes and insight into the understanding of biological processes. Single-particle tracking allows for direct visualization of protein–DNA kinetics, however, identifying whether a molecule is bound to DNA can be non-trivial. Further complications arise when tracking molecules for extended durations in processes with slow kinetics. We developed a machine learning approach, termed Bound2Learn, using output from a widely used tracking software, to robustly classify tracks in order to accurately estimate residence times. We validated our approach *in silico*, and in live-cell data from *Escherichia coli* and *Saccharomyces cerevisiae*. Our method has the potential for broad utility and is applicable to other organisms.

## INTRODUCTION

Quantitative information regarding the kinetics of a protein provides valuable insight into the behaviour of the protein, as well as its relationship with other proteins if it is part of a complex. This in turn may inform on the activity of the protein. The residence times of DNA-bound proteins (DBP) can reveal important details on basic cellular processes such as transcription, DNA repair and DNA replication, at the timescales at which they operate (1–7). This is true for proteins that bind directly at sites on DNA (such as initiator proteins, repair proteins, and chromatin remodellers) and indirectly as part of complexes that bind or translocate on DNA (such as the DNA replication complex (replisome) and RNA polymerase (RNAP)) (1,8).

Recent advances in fluorescence microscopy have allowed us to study protein kinetics directly in living cells, with the most common techniques used being single-particle tracking (SPT), fluorescence recovery after photobleaching (FRAP), and fluorescence correlation spectroscopy (FCS) (3,4,8–13). SPT has the particular advantage of being able to directly observe protein behaviour, allowing for a wealth of information to be extracted from the images, both qualitatively and quantitatively (14,15). Typically, SPT has been used to determine binding kinetics of DBP with very fast kinetics (hundreds of milliseconds to a few seconds), by using capture rates of few to tens of milliseconds. However, many processes operate on much longer timescales posing issues for SPT, including photobleaching and unreliable tracking of single-molecules.

**Figure 1.**
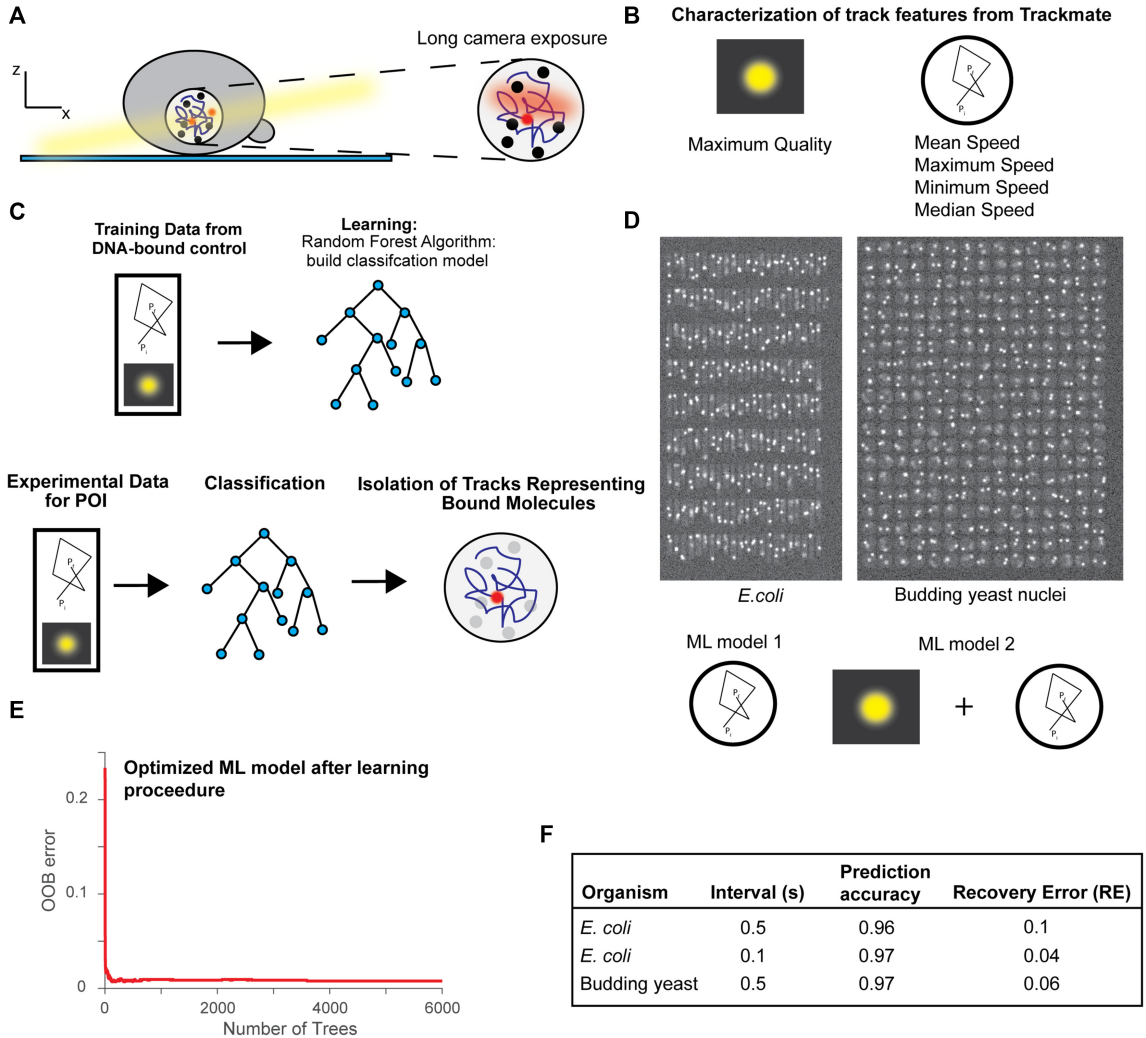
Approach to isolate DNA-bound molecules. (**A**) Diagram of experimental setup to do single-molecule tracking with photoactivatable/photoconvertible fluorophores to calculate residence times on DNA. (**B**) Variables from Trackmate used to predict if a track represents a genuine bound molecule. (**C**) Illustration of the general procedure for ML and how it can be used to predict tracks from experimental data for POI. (**D**) Top: Example images from simulations of single-molecule movies representing *E. coli* cells and budding yeast nuclei. Bottom: Illustration of the variables used in ML models 1 and 2. (**E**) Example of plot to help tune hyperparameters to minimize OOB error. (**F**) Test data results for different ML models.

To bypass these issues, a useful approach is to use long-exposure times to blur out diffusing molecules, in combination with stroboscopic illumination to minimize photobleaching (Figure 1A). Nonetheless, multiple yet unresolved issues continue to complicate the analysis of this approach. Intensity fluctuations caused by the molecule moving out of focus and photophysics of the fluorophores result in fragmentation of tracks (1,16). Reducing the threshold intensity for spot localization can compensate for this at the cost of introducing false positives. A second common problem is the incorrect assignment of diffusive molecules as DNA-bound, despite motion blurring and tracking parameters to select for only bound molecules. This requires further filtering steps so that only tracks representing true DNA-bound proteins are included in the analysis (1,2,17,18). In contrast to complications associated with tracking algorithms and automated analysis, the user can typically distinguish immobile DBP when looking at the raw images under these imaging conditions as they appear to wiggle around a fixed point. This led us to the idea that a machine learning approach would be able to accurately classify the DNA-bound state of a protein.

Here, we provide a user-friendly and robust method called Bound2Learn to determine residence times of DBP using machine learning and SPT in live *Escherichia coli* and *Saccharomyces cerevisiae* (budding yeast), which can be easily extended to other organisms. This approach removes some of the variability in parameter setting and is less sensitive to track fragmentation than other approaches. Hence, we believe it will be particularly useful for the study of protein–DNA binding kinetics with time constants ranging from tens of seconds to minutes.

## MATERIALS AND METHODS

### Computer simulations of single-molecule timelapses

Computer simulations of images were written in Python 3.6.

First, a 5000 × 5000 array was constructed as an image. Each array element was 10 nm, therefore the total size was meant to represent 50μm x 50μm. Cells were placed in a grid-like pattern to prevent overlap and given a low intensity value of around 0.2 per array element to represent cell autofluorescence. *E. coli* cells were modelled as 3D rectangles, with average width of 0.7 μm, and an average length of 3 μm., while budding yeast nuclei were modelled as spheres of average diameter 2 μm. For each cell, unless stated otherwise, two fluorescent molecules were assigned such that their initial locations were confined to the interior of their respective cell. A weighted sampling method was used to assign their initial diffusive state.

To model fluorescent spots, a spot intensity was assigned at the center of the molecules and using a standard deviation of 130 nm, the intensity was spread across the region using a Gaussian filter. To represent out-of-focus spots in the budding yeast simulation, the standard deviation used was increased by 5% for every 150nm increase/decrease in z position of the molecule's position. For example, from ±150 nm from the z-origin of 0, the molecule was assumed to be in focus. However, if the molecule moved to a |*z* position| of >150 nm but less than 300nm, its standard deviation was increased by 5%. After the Gaussian filter, Poisson shot noise at each element was added by sampling from a Poisson distribution with mean equal to the initial intensity value at that element.

Kinetics for individual molecules were determined using transition matrices: one for the photophysics (e.g. photobleaching), and the other for transitions to different diffusive states. We determined the probability of transition using a time step (τ) of 5ms. We omitted photoblinking as the lower laser powers used in long-exposure experiments typically do not cause it. This simplified the photophysics transition matrix to:

**Table:** 

	*F*_state_(*t* + τ)	*B*_state_(t+τ)
*F*_state_(*t*)	1 – CDF_bleach_	CDF_bleach_
*B*_state_(*t*)	0	1

where the *F*_state_ is the fluorescent state, *B*_state_ is the irreversible photobleached state and CDF_bleach_ is the probability of photobleaching within τ, calculated from the cumulative distribution function (CDF) of an exponential distribution with a specified mean bleach time. If the molecule went to the *B*_state_, it was removed from subsequent iterations.

The transition matrix for transitions to different diffusive states was:

**Table:** 

	Mobile_state_ (t+τ)	Bound_state_(t+τ)
Mobile_state_(*t*)	1 – CDF_search_	CDF_search_
Bound_state_(*t*)	CDF_bound_	1 – CDF_bound_

where Mobile_state_ represents the state with *D* = *D*_mobile_, and Bound_state_ represents the state with *D* = *D*_bound,_ CDF_search_ is the probability that the molecule switches to the Bound_state_, based on an exponential distribution with a specified mean search time, and CDF_bound_, is the probability that the molecule switches to the Mobile_state_ based on an exponential distribution with a specified mean bound time. In the case of the heterogeneous population of bound molecules, a second Bound_state_ was added with a different bound time.

From the transition matrices and the respective probabilities, we would select the next state using sampling from a multinomial distribution with the weights given by the probabilities.

To simulate molecule movement, the step size in each *x*, *y*, *z* direction was picked from a Gaussian distribution with variance 2*Dτ*^α^, and α = 1 for the mobile fraction, and 0.4 for the bound fraction to represent subdiffusive behaviour of genomic loci, due to the Rouse model of DNA polymer motion, although we do acknowledge that loci in *E. coli* can undergo more ballistic motions (19–21).

After each time step, the sum of the values of 10 × 10 array elements was taken to simulate a 100 nm pixel size and get a 500 × 500 pixel image. Subsequently, camera noise was added by sampling from gaussian distribution with mean = 150 and standard deviation = 20. To obtain images of different exposure times, time steps were integrated (e.g. 100 ms = 5 ms × 20 steps of integration). In the case of time intervals, we allowed molecule movement but no image formation until the next image is taken, e.g. for 1 s interval, a 500 ms image was followed by 500 ms of only molecule movement but no image formation, followed by another 500 ms image.

### Construction of Top2 and TBP HaloTag fusions

Strains used in this study are all from a BY4741 background and are shown in Supplementary Table S5. Construction of strains was done as previously described (22). Both mNeonGreen and HaloTag fusions were preceded by an eight amino acid linker at the 5′ end (sequence: GGTGACGGTGCTGGTTTAATTAAC). Fluorescent fusions were made by PCR amplification from pTB16 or pSJW01 using the primers listed in Supplementary Table S6.

PCNA-mNeonGreen (from YTB31) and the *pdr5Δ::KanMX* deletion (from a haploid sporulated from YTK1414) were combined by mating and the resulting diploid was dissected to isolate strain ZEY098, a haploid with both PCNA-mNeonGreen and *pdr5Δ::KanMX*. ZEY098 was then mated with either a Top2-Halo or Spt-15-Halo (TBP, TATA-Binding Protein) haploid of the opposite mating type, and the resulting diploids were dissected to create ZEY075 and ZEY157 respectively, the haploids with all three markers (HaloTag, PCNA-mNeonGreen and *pdr5Δ*) that were then used for imaging and control experiments. The genotypes of these imaging haploids are detailed in Supplementary Table S5. Growth curves, flow cytometry and western blots were performed on both strains as described previously (22) to ensure the growth and cell cycle of the cells were not disrupted by the insertion of the HaloTag, and to confirm the HaloTag was not being cleaved and that all fluorescent spots seen were the intact fusion protein.

### Single molecule imaging in budding yeast

A single colony from a YPD plate was placed in 5 ml synthetic complete (SC) medium and grown with shaking at 30°C for ∼5–6 h. This culture was diluted by transferring ∼50 μl into 5 ml of fresh S.C and grown overnight at 30°C. The overnight culture was diluted to 0.15 the next day and grown until the optical density (OD) reached 0.30. 1 ml of this culture was spun down for 1 min @ 4000 RPM, and the pellet was resuspended in 500 ul of fresh S.C. Janelia Farms photoactivatable 549 (PA-JF549) was added to the 500 ul culture for a final dye concentration of 50 nM, except for YTK1434-Halo (Histone H3), where a concentration of 10nM was used to compensate for the higher copy number. This culture was placed in a thermomixer at 30°C and 500 RPM for 40 min. After incubation, three wash cycles using fresh SC were done to wash away unbound dye. After the final wash step, the pellet was resuspended in 50 μl of SC, and 3 μl of the culture was placed on an agarose pad consisting of SC and Optiprep (Sigma), within a Gene Frame (Thermo Scientific). The pad was made by taking a 2% agarose Optiprep mixture (0.02 g in 1 ml Optiprep)—that was heated to 90°—and mixing 500 ul with 500 μl 2× SC, resulting in a 1% agarose 30% Optiprep SC mixture. Approximately 140 μl of this mixture was placed within the Gene Frame, with excess being removed with a KimWipe. Prior to imaging, we waited ∼15 min to let any unbound dye be released.

Coverslips were cleaned with the following steps: (i) place in 2% VersaClean detergent solution overnight; (ii) wash with MilliQ water 3×; (iii) sonicate in acetone for 30 min; (iv) wash with MilliQ water 3×; (v) place in methanol and flame coverslips using Bunsen burner; (vi) place in plasma etch plasma oven for 10 min.

Microscopy was done at 23°C, on a Leica DMi8 inverted microscope with a Roper Scientific iLasV2 (capable of ring total internal reflection fluorescence (TIRF)), and an Andor iXON Ultra EMCCD camera. An Andor ILE combiner was used, and the maximum power from the optical fiber was 100 mW for the 405 nm wavelength, and 150 mW for the 488 nm and 561 nm wavelengths. The iLasV2 was configured to do ring highly inclined and laminated optical sheet (HILO), for selective illumination and single-molecule sensitivity. Metamorph was used to control acquisition. A Leica HCX PL APO 100×/1.47 oil immersion objective was used, with 100 nm pixel size. Any z-stacks were doing using a PInano piezo Z controller.

Single-particle photoactivated localization microscopy (sptPALM) experiments were performed by activating molecules with low power (0.5% in software) 405 nm light to photoactivate ∼1 molecule/cell, followed by stroboscopic, long-exposure (500 ms) illumination with 561 nm light (5% in software) to image primarily bound molecules. A brightfield image and a z-stack of 6 μm (0.3 μm step size) in the 488 nm channel, was taken before and after each timelapse, to ensure normal cell health and to find nuclei.

### Tracking analysis

Tracking was done with Trackmate (23). Spots were localized using the Laplacian of Gaussian (LoG) method, with an estimated spot size of 2.5 pixels, with the exception of the *E. coli* experimental data where it was set to 5 pixels. The intensity threshold was set a bit lower to prevent track fragmentation due to intensity fluctuations. The linear assignment problem (LAP) algorithm was used to form tracks with costs on quality ranging from 0.1 to 0.5. We set a gap frame of 1 to allow temporary disappearance of the molecule, and track merging and splitting was allowed in cases where multiple molecules crossed paths with one another.

To isolate tracks found only in cells/nuclei, we used the binary images to locate tracks whose mean positions coincided with values of 1 in the binary image.

### Machine learning and tracking analysis

All machine learning and subsequent analysis for estimation of residence times was done using Matlab.

To construct training data sets, we had binary classification, with a value of 0 assigned to false positive/diffusing molecule, and a value of 1 to a track representing a bound molecule. We manually looked at the raw image data to determine if the molecule appeared immobile.

For the learning procedure, the ‘TreeBagger’ function in Matlab was used, representing the random forest algorithm. The hyperparameters that were adjusted were: InBagFraction (representing the fraction of the training data given to each tree), MinLeafSize (minimum leaf size), NumPredictorstoSample (number of predictors to sample at random at each node), and NumTrees (the number of trees to construct). InBagFraction was adjusted to make the models generalizable: giving a small sample to each tree will prevent overtraining; however, if the training data was strongly biased towards one class (e.g. mostly bound), then a higher fraction was used to ensure the other class was still being represented during the learning procedure. MinLeafSize was set to 50 for most models, which represents a large enough size to prevent overfitting (since the trees are not unnecessarily extensive), while small enough for trees to have layers and have strong predictive performance. NumPredictorstoSample was set to 2, as for a case of 4–5 variables used during the learning procedure, it provides a balance of accuracy and generalizability. NumTrees was selected until the OOB error was stable and at a minimum (generally over 6000 trees). Too few trees results in poor predictive performance while a large number of trees increases computational time, and may provide no additional benefit after a certain point. These parameters were adjusted until the best OOB error was achieved and performed well on test data, when applicable.

For GMM fitting, the expectation-maximization (EM) algorithm was used.

After the final classification, we analyzed the tracks to extract residence times. We fit the track durations of the resulting tracks with a truncated exponential model, to compensate for discarding short duration tracks, using maximum likelihood estimation (MLE) through Matlab's ‘mle’ function, to calculate the mean track duration.

$$\begin{equation*}PDF = \left( {\frac{1}{\tau }} \right){e^{\frac{{ - \left( {x - L} \right)}}{\tau }}}\end{equation*}$$

where $\tau$ is the mean track duration, and $L$ is the truncation point. For photobleaching controls, this was equivalent to estimating the mean bleach time.

The 95% confidence intervals were calculated by bootstrapping 1000 samples.

In cases where the experimental data was taken with a longer time interval than the photobleaching control, we used the following equation to calculate $Tbleach\;$(1,24):

$$\begin{equation*}{T_{bleach}} = \frac{{{T_{int}}}}{{{T_{exp}}}}\left( {{T_{bleachAFAP}}} \right)\end{equation*}$$

where ${T_{int}}$ and$\;{T_{exp}}$ are the time interval and exposure, respectively, used to acquire the data, while ${T_{bleachAFAP}}$ is the photobleaching time for data collected with continuous exposure, which in in the case of the data used here, would represent 500 ms interval

Bound times were calculated using the following equation, after combining data from multiple experiments collected with the same time interval:

$$\begin{equation*}{T_{bound}} = {T_{track}}*{T_{bleach}}/\left( {{T_{bleach}} - {T_{track}}} \right)\end{equation*}$$

To calculate the errors on the estimate, we performed bootstrap sampling on the track durations to the following equation:

$$\begin{eqnarray*} \left( {\frac{1}{{{T_{track}}}}} \right)\;{e^{ - t/{T_{track}}}} &=& \left( {\left( {\frac{1}{{{T_{bound}}}}} \right) + \left( {\frac{1}{{{T_{bleach}}}}} \right)} \right)\nonumber \\ &&\times\,{e^{ - \left( {\left( {\frac{1}{{{T_{bound}}}}} \right) + \left( {\frac{1}{{{T_{bleach}}}}} \right)} \right)t}} \end{eqnarray*}$$

With 10% variation allowed for the ${T_{bleach}}\;$estimate, in order to obtain biologically sensible results.

To check for two-exponential mixtures, the track durations were fit with the following two-exponential model:

$$\begin{equation*}p\left( {\frac{1}{{{\tau _1}}}} \right){e^{\frac{{ - \left( {x - L} \right)}}{{{\tau _1}}}}} + \left( {1 - p} \right)\left( {\frac{1}{{{\tau _2}}}} \right){e^{\frac{{ - \left( {x - L} \right)}}{{{\tau _2}}}}}\end{equation*}$$

where ${\tau _1}$ = (*Tbleach + Tbound_γ_*)/(*Tbleach* *Tbound_γ_), ${\tau _2}$= (*Tbleach + Tbound_ψ_*)/(Tbleach * Tbound_ψ_), $p$ is the mixture proportion, and $L$ is the truncation point.

The lower and upper bounds on the two binding timescales were 0.0001 and 6000 s, respectively, while allowing for a 10% variation in the bleaching estimate.

To check for overfitting and to identify whether the two-exponential model significantly fit the data better, we used the BIC test and the Loglikelihood ratio (LLR) test, as described in (1). We looked for cases when the two-exponential model estimates did not simply return the lower and/or upper bounds as this would indicate that no physically sensible solution was found.

### Diffusion coefficient estimation

Diffusion coefficients were estimated from individual tracks by calculated the slope of the time lag (τ) vs mean squared displacement (MSD) curve. The equation used was modified from (25):

$$\begin{eqnarray*} && 4*Dapp*{\tau ^\alpha } \nonumber \\ && + \left( {4*{b^2} - 8*\left( {\frac{1}{6}} \right)*\left( {\frac{{Texp}}{{Tint}}} \right)*D*Tint} \right) \end{eqnarray*}$$

where $Dapp$ is the apparent diffusion coefficient, α is the anomalous diffusion constant, and $b$ is the static localization error. We used this equation to correct for dynamic localization error due to the molecule moving within the exposure time, which in our case was quite long.

To classify tracks using their Dapp estimates, we performed GMM fitting on the distribution of Dapp, with two components, and clustered tracks according to their Dapp to assign them as either being in the bound state vs diffusing/noise. Clustering was done by first calculating posterior probabilities to each track (the probability it belongs to a diffusive state given its Dapp), and then selecting the diffusive state with the maximum posterior probability.

To gain a better estimate of the diffusion coefficient for Top2, TBP, and Histone H3, we performed a weighted, least -squares fit using the MSD values averaged over the tracks, after they were classified by Bound2Learn. We did not include tracks with a goodness of fit (gof) score of less than 0.70 from the previous step, given the noisiness of some of the MSD curves. We also included lower and upper bounds on the estimates to ensure physically sensible estimates, and in cases where on the estimates was the lower or upper bound value, we discarded data points at higher τ values. This is a known issue given there are fewer data points in the calculation of MSD at the higher τ values leading to more noisier traces.

## RESULTS

### Random Forest for single-molecule tracking classification

Machine learning (ML), including its branch of deep-learning, is a powerful tool for image analysis and classification, with the most common implementation being supervised learning, whereby a labelled training data set is given to the ML algorithm, which then builds a model for subsequent classification (26,27). Our motivation arose from recognizing the limitations associated with automated detection and tracking, and our desire to develop a classification method to compensate for these limitations. We recognized that a known DBP (e.g. histone H3) could be used to construct a training data set manually, for how DBP in general should move and how single-molecules should look like in images, from which we can build a ML model. We can also use this control as a photobleaching control—if it exhibits stable binding—when we estimate the residence time for our DNA-bound protein of interest (POI) (1).

We used Trackmate, a freely available plugin in Fiji, to track molecules and used variables from the tracking output that we believed to be good predictors of single-molecule DBP and reduced the cross-validation error (CVE) (Figure 1B) (23). To predict whether a molecule moves like a DBP we used the following variables from the tracking output: mean speed, maximum speed, minimum speed, and median speed. For predicting whether the track represented a genuine molecule, we used the maximum quality variable—a parameter corresponding to the intensity of the molecule as well as its shape (23). Classification of the track was done manually by looking at the raw image data, with binary classification: 0 for a diffusing molecule or noise, and 1 for a track representing a genuine immobile, DNA-bound molecule. We chose to use the random forest algorithm to construct a model from the training data, as it is accurate, tolerant of noise, and less prone to overfitting (28) (Figure 1C). The various parameters used to construct the different random forest models are listed in Supplementary Table S1. We used this model to classify tracks for our POI (Figure 1C), and subsequently determine their residence time after correcting for photobleaching.

We first tested our approach using computer simulations of single-molecule timelapses of *E. coli* and nuclei of budding yeast (see Materials and Methods). The main difference between the two simulations, aside from cell shape, was that the budding yeast simulation had a distortion in the shape of the molecule based on its position in z, to model the point spread function (PSF) and emulate the molecule going out of the focal depth of the objective. Under our experimental conditions, *E. coli* cells have diameters of 0.7 μm, which is not far from the estimated focal depth of high numerical aperture objectives commonly used in single-molecule studies (∼0.4 μm), so we assumed molecules to be in focus, regardless of their z position (1). We first constructed training data sets from simulated data with 500ms exposure, no time interval, and a mean bleach time of 10s (Supplementary Table S2). We had a stable, bound fraction (diffusion coefficient (*D*) of *D*_bound_ = 0.005 μm^2^/s, bound time >>> bleach time), along with a mobile fraction (*D*_mobile_ = 0.5 μm^2^/s) (Figure 1D). We chose a *D*_mobile_ of 0.5 μm^2^/s, as it represents an appropriate lower limit for a diffusing molecule and is on the order of previously used values used in simulations to benchmark measurements of DBP (3,18,29). The value for *D*_bound_ was selected for similar reasons.

We only considered tracks with ≥4 localizations, as it is difficult to discern the state of the molecule for shorter tracks, and throughout the rest of the sections we used a low intensity threshold for localization during analysis to prevent track fragmentation, with the ML approach meant to discard any false positives of DBP (3,18). In order to make use of a single training data set, instead of constructing multiple training data sets for classifying data collected under different conditions, we constructed two models: ML model 1 only has the speed variables and ML model 2 has the speed variables along with maximum quality (Figure 1D). We calculate the mean of ‘mean speed’ as well as the mean of ‘maximum quality’ from the tracks classified as being bound in the training data and used these values to scale the speed and quality variables, respectively, for data collected with different time intervals and/or illumination intensities (Supplementary Figure S1).

The entire procedure for the classification of tracks for a POI is as follows:

1. We performed a two-component Gaussian mixture model (GMM) fit on the log of mean speed, of all the tracks, and the component with the lowest mean is selected as representing the immobile molecules. This step does not need to be robust as it is only used to do some initial filtering for subsequent steps.
2. Using this value, we scale the speed variables accordingly, using the mean of ‘mean speed’ calculated from the training data, as follows: scale factor_speed_ = mean (mean speed_training_)/mean(mean speed_boundPOI_). This helps to ensure the ML models can classify tracks obtained from different time intervals than the training data. We then run the tracks through ML model 1 for initial filtering.
3. From the resulting tracks, we calculate the mean of ‘maximum quality’ using a two-component GMM, similar to step 1 except selecting the component with the higher mean and use the same variable from the training data to scale the quality variable, similar to the previous step. We then run the tracks through ML model 2 for final classification.

To assess how well the procedure worked, we quantified both the accuracy (proportion of tracks accurately predicted to be bound), and the recovery error (RE) (the fraction of tracks known to be representing bound molecules that were missed by the classification procedure). While high classification accuracy is important to ensure accurate estimation of residence time, we also wanted to make sure that the recovery error is small, as single-molecule studies are often plagued by low sample sizes that reduce confidence in the estimate (1,14). In addition, the out-of-bag (OOB) errors, equivalent to the CVE for random forests (28), were estimated for each ML model (Supplementary Table S1). The hyperparameters during the learning procedure (e.g. number of trees, bag fraction, number of variables to sample) were optimized to give the lowest OOB error and best results on test data (Figure 1E). On test data, we obtained an accuracy of 0.96 and RE of 0.10 for the *E. coli* simulation, and an accuracy of 0.97 and RE of 0.06, for the budding yeast simulation (Figure 1F).

### Accurate estimation *in silico* of residence times under different conditions

**Figure 2.**
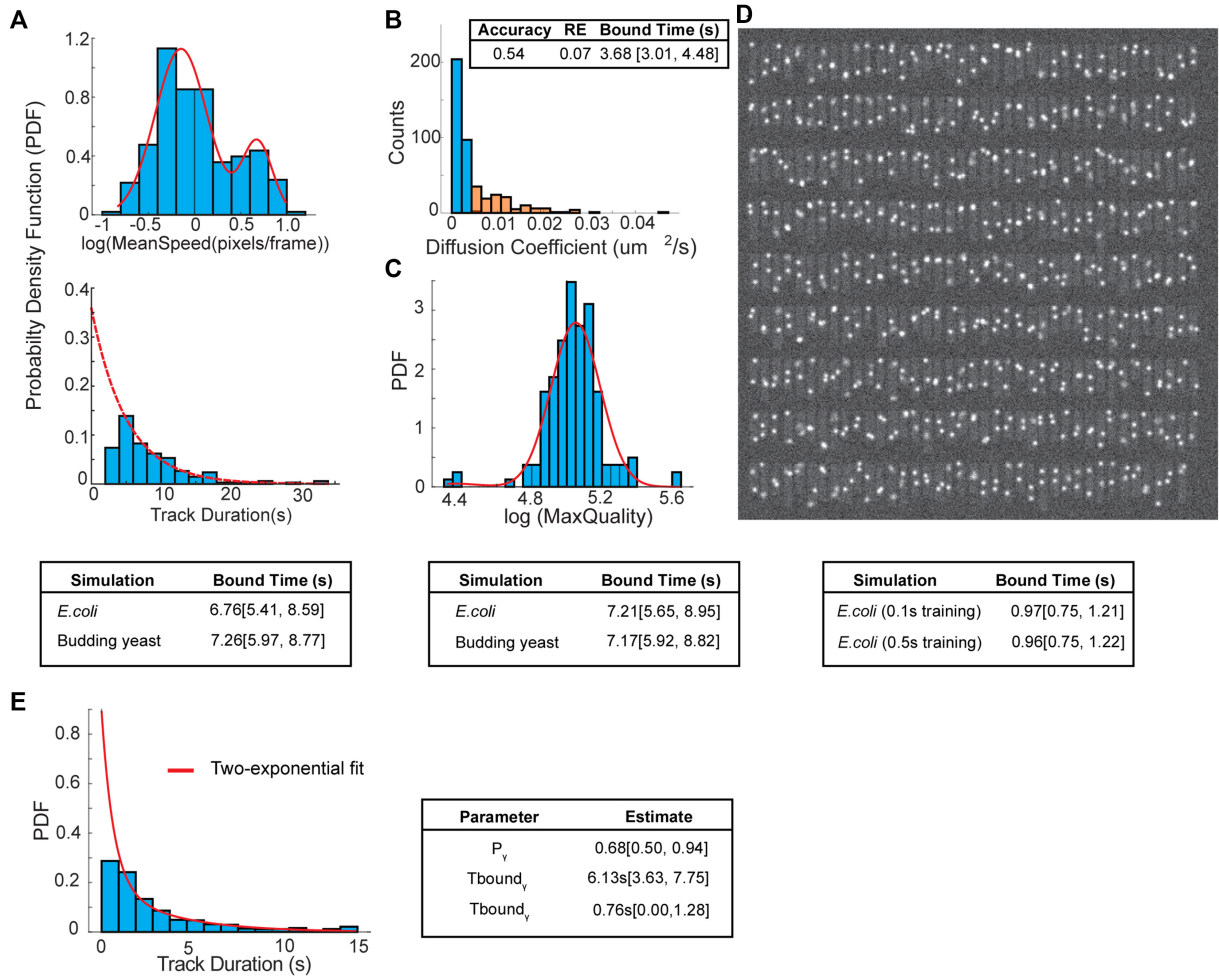
Estimating residence times over a range of conditions. (**A**) Top: Diagram illustrating the GMM fit to determine the mean of mean speed for 1 s interval data, which can be used to rescale the speed variables. Bottom – Estimates of residence times in both simulations (*n* = 169 tracks for *E. coli* and *n* = 232 tracks for budding yeast). (**B**) Distribution of apparent diffusion coefficients from *E. coli* simulation, with GMM fitting, followed by clustering to isolate tracks representing bound molecules (blue) and diffusing/noise (orange). *n* = 291 tracks. (**C**) Top: Diagram illustrating the GMM fit to maximum quality values from a data set with lower fluorescent intensities. Bottom: Estimates of residence times from simulated data representing poorer image quality (*n* = 159 tracks for *E. coli* and *n* = 227 tracks for budding yeast). (**D**) Top: Example of image from simulated 100 ms timelapse in *E. coli*. (**E**) Two-exponential fitting to a data set with heterogeneous population of bound molecules (*n* = 508 tracks). For all estimates, 95% confidence intervals are shown.

Next, we tested how well the ML models would work on simulations of image data with different time intervals and spot intensities (Materials and Methods, Supplementary Table S2). First, we tested on data with a 1 s time interval and the same spot intensities as the training data set (Supplementary Videos S1 and S2). The mean residence time was set to 8 s, while the bound fraction was set to 0.5. As Figure 2A (top) illustrates, we first calculate the mean of ‘mean speed’ of the bound population, in order to scale the speed variables. After the final classification step, for both the *E. coli* and budding yeast simulations, we were able to obtain a mean residence time estimate (∼7 s) that was in close agreement with the 8s set in the simulation (Figure 2A (bottom)). In addition, the accuracy values were 0.93 and 0.96, with recovery errors of 0.11 and 0, respectively, for the *E. coli* and budding yeast simulations (Supplementary Table S3).

With the *E. coli* simulation, we also tested the accuracy and RE by using a more traditional approach of estimating apparent diffusion coefficients (Dapp) through mean-squared displacement (MSD) analysis, GMM fitting to determine diffusive fractions, and assigning individual tracks to a diffusive state using a cluster method (Materials and Methods). We observed that there was a large spread in the apparent diffusion coefficients, consistent with the difficulty of obtaining precise estimates from single tracks using MSD analysis, given that they are inherently noisy (25) (Figure 2B). Furthermore, the accuracy and RE using this approach was 0.54 and 0.12, respectively, with a bound time estimate much lower than 8s, suggesting a significant fraction of tracks resembling diffusing molecules or noise were being classified as bound (Figure 2B). This suggests that while it is possible that mean values of the Dapp for different states may be accurate, due to precision issues from MSD analysis, assigning individual tracks to different states based solely on their Dapp can be error prone. We then tested whether we could obtain an accurate estimate in a situation representing poorer image quality, by lowering the integrated spot intensity from 3000 to 2000. As Figure 2C (top) illustrates, we use the GMM fit on the max quality values to find an appropriate scaling factor. After classification, we find that we can still recover an estimate of the residence that is within error of the known value, for both simulations, with accuracy values and REs similar to the previous condition (Figure 2C, Supplementary Table S3). In contrast, the MSD analysis approach on the same data resulted in a lower accuracy of 0.79, with a RE of 0.096, and bound time estimate of ∼5 s (Supplementary Table S3).

While a long exposure of 500 ms helps to blur out diffusing molecules, it also restricts the temporal resolution achievable, and makes it difficult to obtain precise estimates for faster processes, as fewer tracks will remain if using a track localization acceptance threshold. Therefore, we constructed a training data set with 100 ms exposure [accuracy and RE on test data was 0.97 and 0.06, respectively (Figure 1E)], and tested on data with a mean residence time set to 1s while the bleach time for this condition was set to 2 s (20 frames) (Figure 2D, Supplementary Video S3). Once again, we were able to obtain an estimate (∼0.97 s) that was in agreement with the 1s residence time, although with lower accuracy and higher recovery errors than with 500ms, as one would expect under conditions where the separation between two diffusive states is more difficult to discern. (Figure 2D). We tested this by changing *D*_mobile_ to 5 μm^2^/s, and found the errors were drastically reduced, with an accuracy of 0.98 and RE of 0.08 (Supplementary Table S3). We then asked whether the ML models built with the 500 ms training data can be used to classify the 100ms data, and surprisingly, we found that it still performed well (Figure 2D), with comparable errors to the 100 ms ML models (accuracy = 0.86 and RE = 0.16 with 500 ms ML model, versus accuracy = 0.85 and RE = 0.18 with 100 ms ML model) (Supplementary Table S3).

We also found that we could get accurate estimates of the residence times of a heterogeneous population of bound molecules, where two distinct binding regimes were present: mean Tbound_γ_ set to 7 s while mean Tbound_ψ_ set to 1 s (Figure 2E). We note that for the simulation representing a heterogeneous population of bound molecules, we had to change the bleach time to 10 s, in order to recover the two binding times. As others have alluded too, detecting multiple populations is highly dependent on acquisition settings (30). These results show that one can use ML models constructed from a single training data set and use them for accurately classifying tracks obtained from data collected under widely different conditions, along with obtaining accurate residence times of homogeneous and heterogeneous bound populations. It is important to highlight that this analysis does not require additional steps to identify appropriate thresholds to filter out noise and/or diffusing molecules, which can be time-consuming and a source of heterogeneity in the analysis.

**Figure 3.**
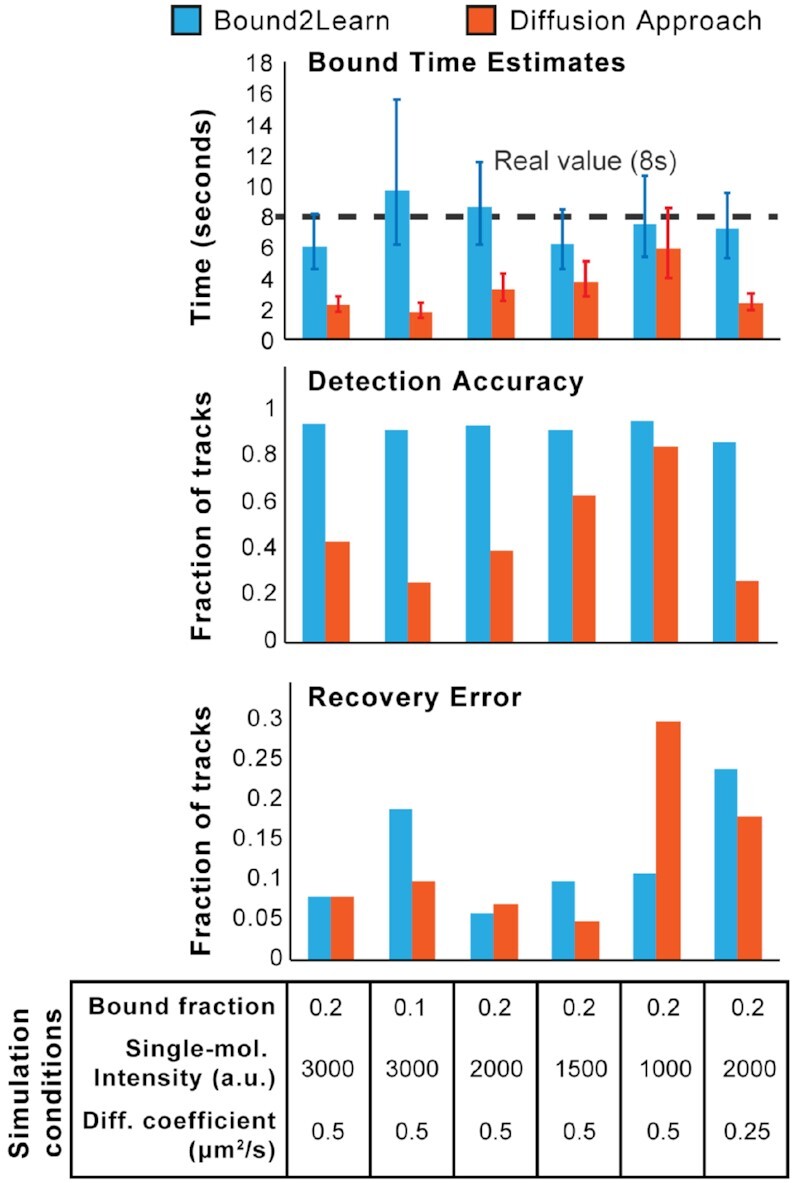
Testing Bound2Learn under challenging simulation conditions for tracking. Simulations were done based on *E. coli* cells and the same tracking analysis parameters were used across all conditions. The input bound time was 8 s while the bleach time was 20 s. Images represented 500 ms exposure with a 1 s time interval. Error bars for bound time estimates represent 95% confidence intervals.

We also characterized our approach across a range of different simulation conditions and compared it to the commonly-used MSD analysis approach for classifying states of molecules from SPT (Figure 3) (2,11,29). Here we tested the performance of our approach in conditions where we reduced the proportion of DNA-bound to diffusive molecules, the signal to noise ratio of the spots, and the diffusion coefficient of diffusive molecules. All these conditions were expected to complicate the accurate estimation of the bound times. We found our approach consistently out-performed the MSD analysis approach, and achieved estimates for the bound time in close agreement with the input of 8 s. The Bound2Learn approach was able to perform robustly even in conditions where the intensity of the spots was one third of the optimal intensity (Figure 3, condition 5). Compared with the Bound2Learn approach, the MSD analysis approach had a much lower detection accuracy, but with similar RE, suggesting that the MSD approach resulted in a higher number of false positives. We note that this does not imply that the MSD approach is poor in general for classification, but rather, under tracking conditions meant to prevent track fragmentations, it can have a high classification error. The procedure used for image simulation constrained the lower-bound limit for the diffusion coefficient of diffusing copies, below which molecules may appear to become stuck at the boundaries of the cell. Hence, we did not simulate molecules that diffused slower than 0.25 μm^2^/s. However, this value is roughly half of the slowest diffusive DBPs in *E. coli* (29). Overall, these results make us confident that our ML approach can robustly classify DBP and estimate bound times across a range of conditions.

### Experimental validation in *E. coli*

**Figure 4.**
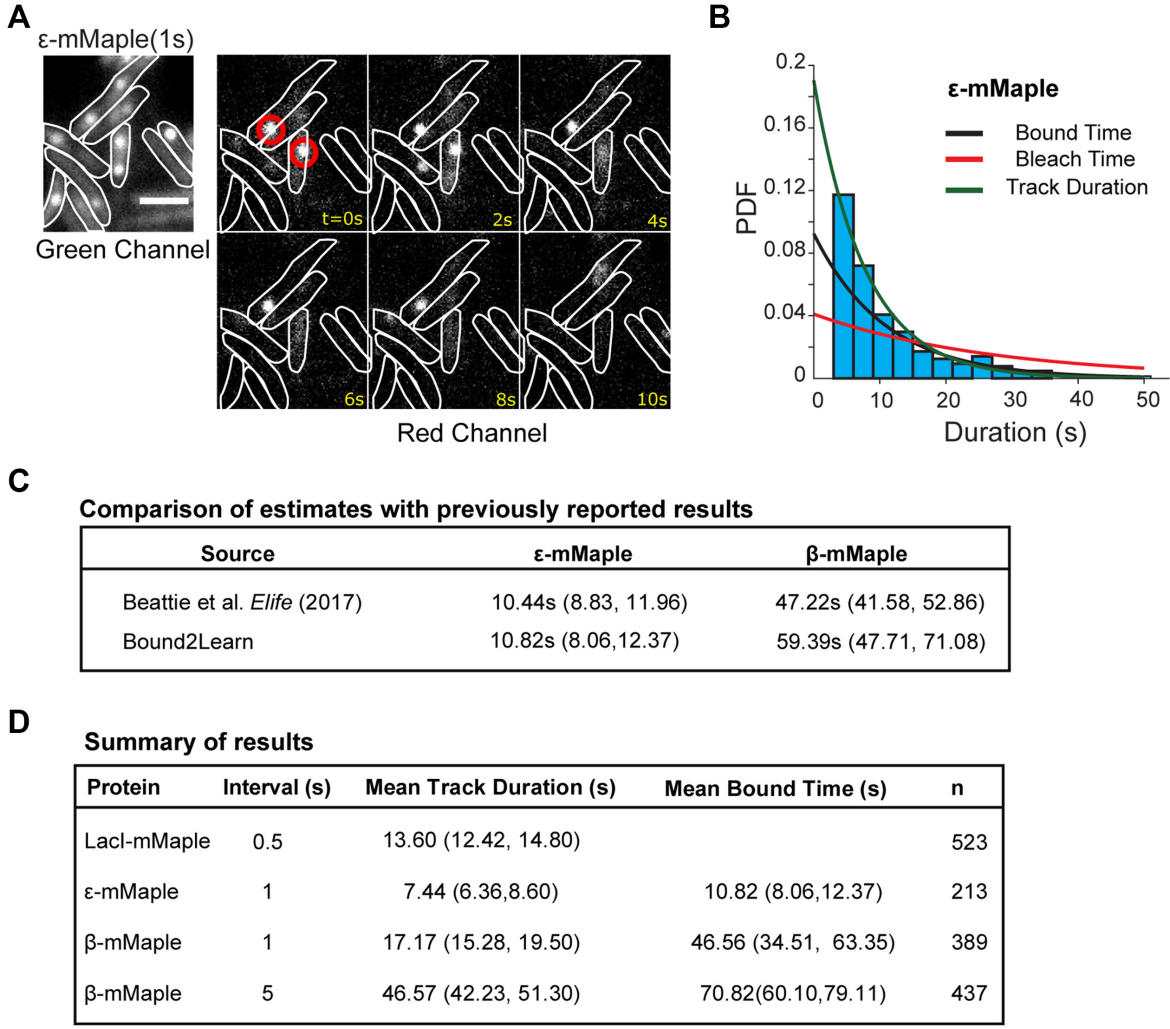
Estimating residence times for PolIII subunit, ϵ. (**A**) Example of ϵ-mMaple timelapse. Red circles indicate molecules that were classified as being bound with Bound2Learn. Scale bar = 2 μm. (**B**) Histogram and fit of track durations from combined data set of ϵ. (**C**) Estimation of residence times and comparison to estimates from (1). Estimate for β was a weighted average calculated from bound times estimated with different time intervals. (**D**) Summary of results showing both mean track duration estimates and mean bound time estimates from combined data sets. 95% confidence intervals are shown next to estimates.

We then determined if Bound2Learn could estimate residence times from imaging of *E. coli*. Unless stated otherwise, estimates were obtained by fitting track durations using a truncated exponential model (we discarded tracks with <4 localizations as they were not reliable) to data from multiple experiments collected under the same acquisition settings to obtain higher statistical confidence (Materials and Methods, Supplementary Table S4). We reanalyzed a subset of the data reported in (1) to test if we could obtain consistent results. For our DNA-bound control we used LacI, a transcriptional repressor, fused to the photoconvertible mMaple, which upon illumination with 405 nm light, converts to a red fluorescent form (31). LacI has been reported to bind stably to the *lacO* array site on DNA (∼5 min), thus making it suitable as a photobleaching control, under our acquisition times (32) (Supplementary Figure S2). Although we had initially used this strain for photobleaching correction in (1), we decided to use it for constructing a training data set as well. The LacI data used for the training data was collected with 500ms exposure and continuous acquisition, resulting an estimated photobleaching time of 13.60 s (Figure 4D).

Once the ML models were constructed, we asked whether we could get an accurate estimate of the residence time for *E. coli* DNA polymerase, PolIII, for which we had previously reported to have a residence time of around ∼10 s, (1). We reanalyzed a dataset from (1) taken of the PolIII subunit, ϵ, also tagged with mMaple (ϵ-mMaple), to determine if Bound2Learn could give similar results (Figure 4A). Our analysis resulted in an estimate of 10.82 s (8.06, 12.37 s), in very close agreement with the previously reported estimate (Figure 4B and C). We also reanalyzed data taken of the DNA binding clamp, β-mMaple, which we previously reported to have a bound time of 47.22 s (1). As mentioned before, stable binding poses an issue for tracking as intensity fluctuations (e.g. molecule moving out-of-focus) can result in track fragmentation (1). Bound2Learn is less sensitive to such intensity fluctuations since it allows the use of lower intensity thresholds during the localization step and still result in accurate classification. We analyzed data collected with 1s and 5s time intervals, and then calculated a weighted average as our final estimate for mean bound time. Our estimate using Bound2Learn was 59.39 s (47.71 s, 71.08 s), which is slightly higher, but still within error, than our previously published estimate (Figure 4C). Use of a different localization and tracking software than the one in our previous publication may also explain part of this difference (1). Our full results are summarized in Figure 4D, showing the estimates for mean track durations and mean bound times for the different proteins, and different time intervals in the case of β_2._ Overall, these results suggest that Bound2Learn robustly estimates residence times of proteins with different binding behaviours in *E. coli*.

### Estimating residence times of topoisomerase II and TATA-binding protein in budding Yeast

We then asked if our approach could be used in live budding yeast. For our photobleaching control and training data construction, we used histone H3 fused to HaloTag (H3-HaloTag), due to its expected long residence time and high bound fraction (33) (Supplementary Figure S2 Table S5). In order to detect the protein, we incubated cells with the cell-permeable photoactivatable (PA) dye, PA-JF549 (34). The experimental protocol was very similar to that described in *E. coli* (1), consisting of the use of highly-inclined and laminated optical (HILO) sheet stroboscopic illumination with 500ms exposure, but with cycles of low-dose 405 nm activation every 40 frames **(**Materials and Methods**)**. To improve image quality with budding yeast, we also used the refractive-index matching media, Optiprep, to minimize light refraction due to the cell wall (35). The mean photobleaching duration estimated, with 1 s interval acquisition, was ∼22 s (Supplementary Table S4, Supplementary Figure S2).

**Figure 5.**
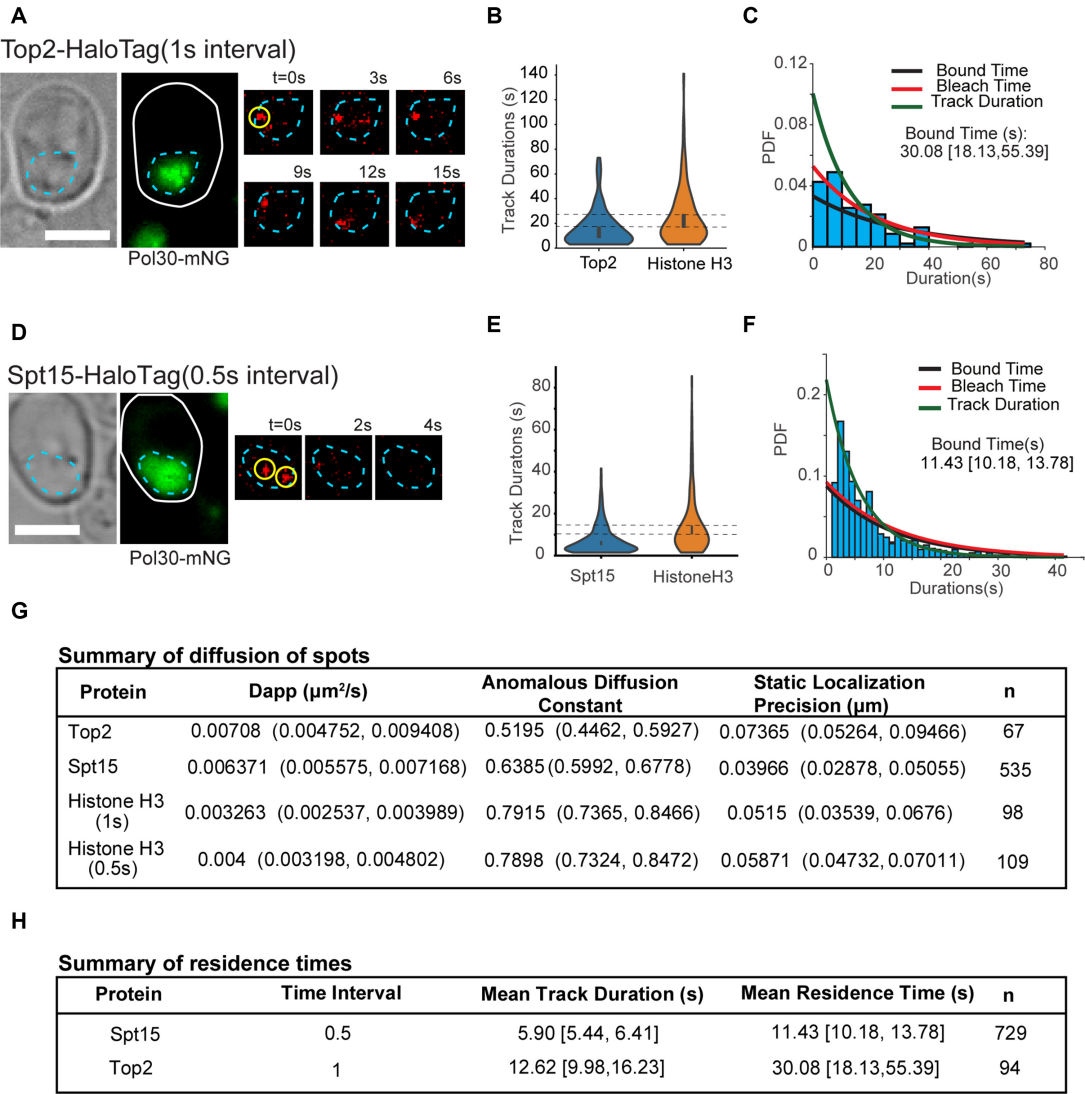
Estimating residence time of Top2 and TBP in budding yeast. (**A**) Example of Top2-HaloTag timelapse after photoactivation. Yellow circle indicates molecule classified as being bound with Bound2Learn. Cell and nucleus outlines are drawn in white and cyan, respectively. Also shown is the SME of a Pol30-mNeonGreen z-stack. Scale bar = 3 μm. (**B**) Violin plots of track durations for Top2 (*n* = 94), and Histone H3 (*n* = 123). Error bars represent 95% confidence intervals. Dotted horizontal lines represent upper and lower bounds of the 95% confidence interval of Histone H3. (**C**) Histogram and fit on Top2 track durations. Note that bin counts for short durations are smaller than expect given that short tracks of <4 localizations were discarded. This was compensated for during the fitting procedure (Materials and Methods). Errors are represented by 95% confidence intervals (**D**) Example of SPT15-HaloTag (TBP) timelapse collected with continuous exposure, after photoactivation. Yellow circles indicate molecules classified as being bound by Bound2Learn. Cell and nucleus outlines are drawn in white and cyan, respectively. Also shown is the SME of a Pol30-mNeonGreen z-stack. Scale bar = 3 μm. (**E**) Violin plots of track durations for TBP (*n* = 729), and Histone H3 (*n* = 242). Error bars represent 95% confidence intervals. Dotted horizontal lines represent upper and lower bounds of the 95% confidence interval of Histone H3. (**F**) Histogram and fit on TBP track durations, along with estimate for bound time. (**G**) Estimates for diffusive properties and static localization errors of ML classified bound tracks, obtained through fitting averaged MSD curves. (**H**) Summary of results showing both mean track duration estimates and mean bound time estimates from combined data sets. 95% confidence intervals are shown next to estimates.

As experimental test, we tagged Topoisomerase II (Top2) with HaloTag (Top2-HaloTag) and to segment nuclei in order to isolate tracks found only in S-phase nuclei, we tagged proliferating nuclear cell antigen (PCNA) with mNeonGreen (Pol30-mNeonGreen) and acquired a z-stack in the green channel prior to acquisition (Supplementary Table S5). Tagging Top2 with HaloTag did not result in any visible growth or viability defect of the cells, or in the degradation of the protein (Supplementary Figure S3). The resulting z-stack went through a smooth manifold extraction (SME) process for better image quality of the nuclei (36). We chose Top2 as mammalian Top2 enzymes have been reported to be dynamic in interphase cells, with FRAP *t*_1/2_ lifetimes of 1.8–10 s (no precise residence time determined since the recovery due to diffusion versus Top2 dissociation was not determined). A more recent study examining Top2A dynamics in mitosis reported a FRAP recovery time that consisted of two *t*_1/2_ lifetimes, with the longer timescale (representing DNA-bound fraction) estimated to be ∼ 30–60 s (13,37). We collected the data under the same acquisition settings as H3-HaloTag. As observed in Figure 5A, we were able to visualize dissociation events of the protein under our acquisition settings. Following tracking analysis and ML classification, we obtained an estimate for the residence time of Top2 of ∼30 s, consistent with dynamic behaviour reported previously in mammalian cells (Figure 5B and C, Supplementary Table S4). We also performed similar experiments in a strain lacking a HaloTagged protein, but incubated with the fluorescent dye, and could not observe any molecules in the timelapses (Supplementary Figure S4). In addition, no bound tracks were obtained after classification with Bound2Learn. Therefore, nonspecific binding of the dye is unlikely to contribute to our estimates. It should be noted that despite slightly poorer spot quality compared to *E. coli* (compare Figure 4A versus Figure 5A), Bound2Learn was able to obtain estimates in this system, suggesting our approach can be used across a range of imaging conditions.

To further confirm that our approach works with single-molecule data from budding yeast, we performed similar experiments with a HaloTag fusion of TATA binding protein (TBP) (SPT15-HaloTag), a general transcription factor, which has been shown in budding yeast to have FRAP recovery times <15 s, and with a minimal immobile fraction, suggesting it is dynamic on DNA, although the exact binding time has not been determined (8) (Supplementary Table S5). We collected the data with continuous exposure (500 ms intervals) rather than with a 1s time interval, to prevent the possibility of TBP binding to neighboring binding sites thereby artificially increasing its estimated residence time. Our data confirms the dynamic behaviour of TBP, as we observed dissociation within a few seconds (Figure 5D, Supplementary Video S4). Few of the spots detected were in S-phase nuclei, indicating that some transcription (e.g. expression of histones) occurs during S-phase (38). Our measured photobleaching time using histone H3 was 12.19 s (Supplementary Table S4). When we quantified the track durations for TBP, we observed a significantly different track duration distribution relative to histone H3, with an abundance of short duration tracks, suggesting most tracks ended due to dissociation of the protein as opposed to photobleaching (Figure 5E, Supplementary Table S4). Our estimate for the mean bound time of TBP, after photobleaching correction, was 11.43 s, consistent with TBP being dynamic on DNA (Figure 5F).

To increase our confidence that the tracks classified as being DNA bound were actually DNA-bound, we tested if they had diffusive properties similar to chromosomal loci. We estimated the apparent diffusion coefficients and anomalous diffusion constants for the different budding yeast proteins used here by fitting the τ versus MSD curve (Materials and Methods) (22). Remarkably, the estimates were not only consistent among the different proteins (Dapp ranging from 0.003263 to 0.007080 μm^2^/s) but were consistent with previously estimated values of chromosomal loci in budding yeast of *D*_app_ ∼0.0025 μm^2^/s and α ∼ 0.5 (20) (Figure 5G). We did notice that histone H3 had slightly different values compared to TBP and Top2, but given that histones are widespread on chromosomes, it is possible that we detected histone H3 molecules bound to chromosomal segments where TBP and Top2 do not bind frequently (e.g. telomeres) (22).

The results from budding yeast, summarized in Figure 5H, show estimates for mean track durations and mean bound times for TBP and Top2. Although our results show that both of them have bound times of several seconds, further experiments need to test the biological relevance of these binding kinetics by, for example, using mutant proteins impaired in different aspects of their function. Despite not causing any evident growth defect, we cannot rule out that the HaloTag may have a negative effect in the function of these proteins. However, our results broadly agree with what has previously been reported for these proteins. Overall, these results show that our approach can accurately determine residence times across a range of binding behaviours in two very different organisms.

## DISCUSSION

Here, we have provided a robust, easy-to-use classification approach to isolate tracks of DBP and estimate residence times from long-capture (motion blurred) single-molecule data. Commonly used approaches to classify bound molecules by applying thresholds on various track traits (MSD analysis, step-size per frame of DBP, PSF, etc.) often are not sufficient for rigorous classification, require additional analysis steps and/or experiments, or may discard too many tracks (1,2,18,25). Our approach bypasses these limitations, without requiring significant technical expertise or computational resources, and can obtain estimates in a rapid manner. This new approach is particularly advantageous for the study of DBPs with residence times ranging from tens of seconds to minutes (where molecules moving out-of-focus is an issue), as has been recently shown to work very well for replication proteins (22). Notably, Bound2Learn permits the use of tracking analysis parameters to prevent track fragmentation (e.g. low intensity threshold for localization) that is prohibited with a MSD analysis approach. Since Bound2Learn functions by differentiating the almost immobile bound copies from the diffusive copies, we expect that our approach may be also adaptable to the study of the binding kinetics of proteins that bind to other cellular structures, such as lipid membranes or the cytoskeleton.

Bound2Learn was able to recover the residence time even in suboptimal signal-to-noise conditions. Despite this observation, a requirement for good image quality is still the main limitation of our approach. Lower intensity will not only lead to lower quality values but also overestimate speed variables for classification due to increased localization error. Similarly, the classification error in our approach increases when the diffusive and DNA-bound fractions have similar mobility, although as demonstrated above Bound-2-Learn should performs robustly under most biologically relevant diffusive values. Another limitation is that constructing training data sets manually can take significant time and choosing the right hyperparameter values for building random forest models requires some expertise; to facilitate this step we provide advice on how to train and tune the model in the methods as well as providing a link to an instructional video. However, our results suggest that once the initial models have been created, they can be used across a range of conditions whether that be different exposure times, time intervals, data quality, and are amenable for use in a high-throughput manner. During this study, we had to adjust our simulation parameters (akin to adjusting experimental conditions) to detect two binding regimes in cases where there was a heterogeneous population of bound molecules, similar to what a recent study has reported (30). Nonetheless, this is not an issue with our classification approach but rather the acquisition settings used (or in our case, simulation parameters), and our results suggest simulations can help identifying the optimal acquisition conditions to use for detecting multiple binding states.

Use of Bound2Learn solves some of the limitations with previous approaches for classification of DNA-bound molecules, demonstrating an advantage of using ML approaches. Future development of this approach should focus on decreasing its sensitivity to image quality. A potential method to doing this may be the incorporation of Deep Learning approaches for the denoising and other pre-processing of the images, an area of image analysis that has had a fast development recently (39). As it stands, we expect that Bound2Learn will improve the analysis of other DNA-binding proteins, and other proteins interacting to relatively immobile structures in the cell. We also expect that this approach will be easily applicable to other experimental models, including mammalian cells.

## DATA AVAILABILITY

Software code and a link to a video on how to use the script are available at https://github.com/Reyes-LamotheLab/Bound2Learn. All data is available upon request.

## Supplementary Material

gkab186_Supplemental_Files

## Notes

Present address: Nitin Kapadia, The Francis Crick Institute, 1 Midland Road, London NW1 1AT, UK.
