# Supplementary material for: Bound2Learn: a machine learning approach for classification of DNA-bound proteins from single-molecule tracking experiments
Source: Nucleic Acids Res. 2021 Mar 21;49(14):e79. doi: 10.1093/nar/gkab186 (PMC8373171; doi:10.1093/nar/gkab186)
Supplement: gkab186_Supplemental_Files [file gkab186_supplemental_files.zip › Supplementary Information.pdf]

Supplementary Information

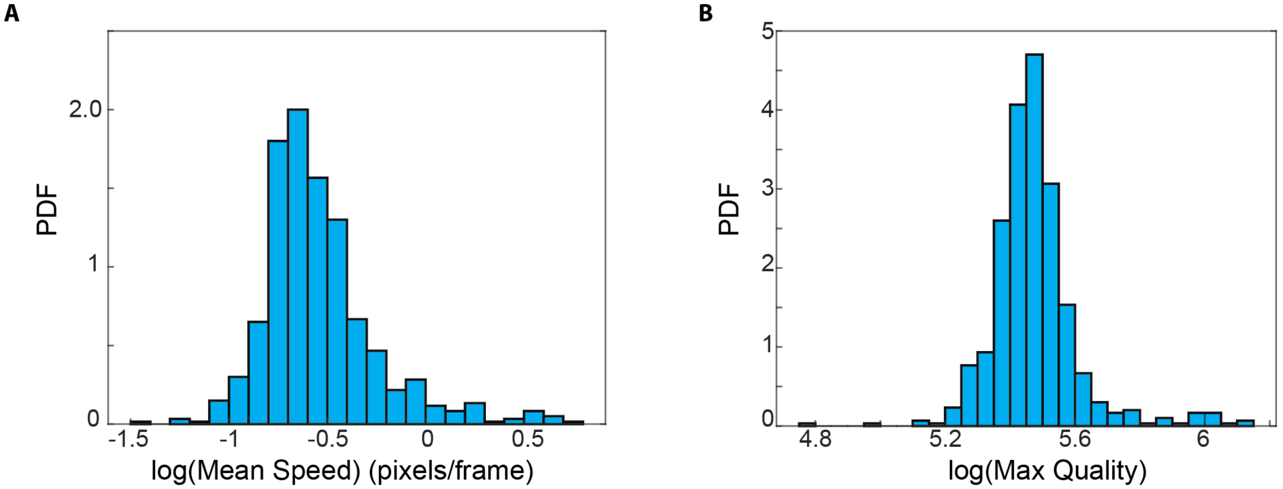

Figure S1. A) Representative distribution of the log(mean speed) values for tracks classified as being bound from training data set (500ms, *E.coli*). B) – Representative distribution of the log(maximum quality) values for tracks classified as being bound from training data set (500ms, *E.coli*).

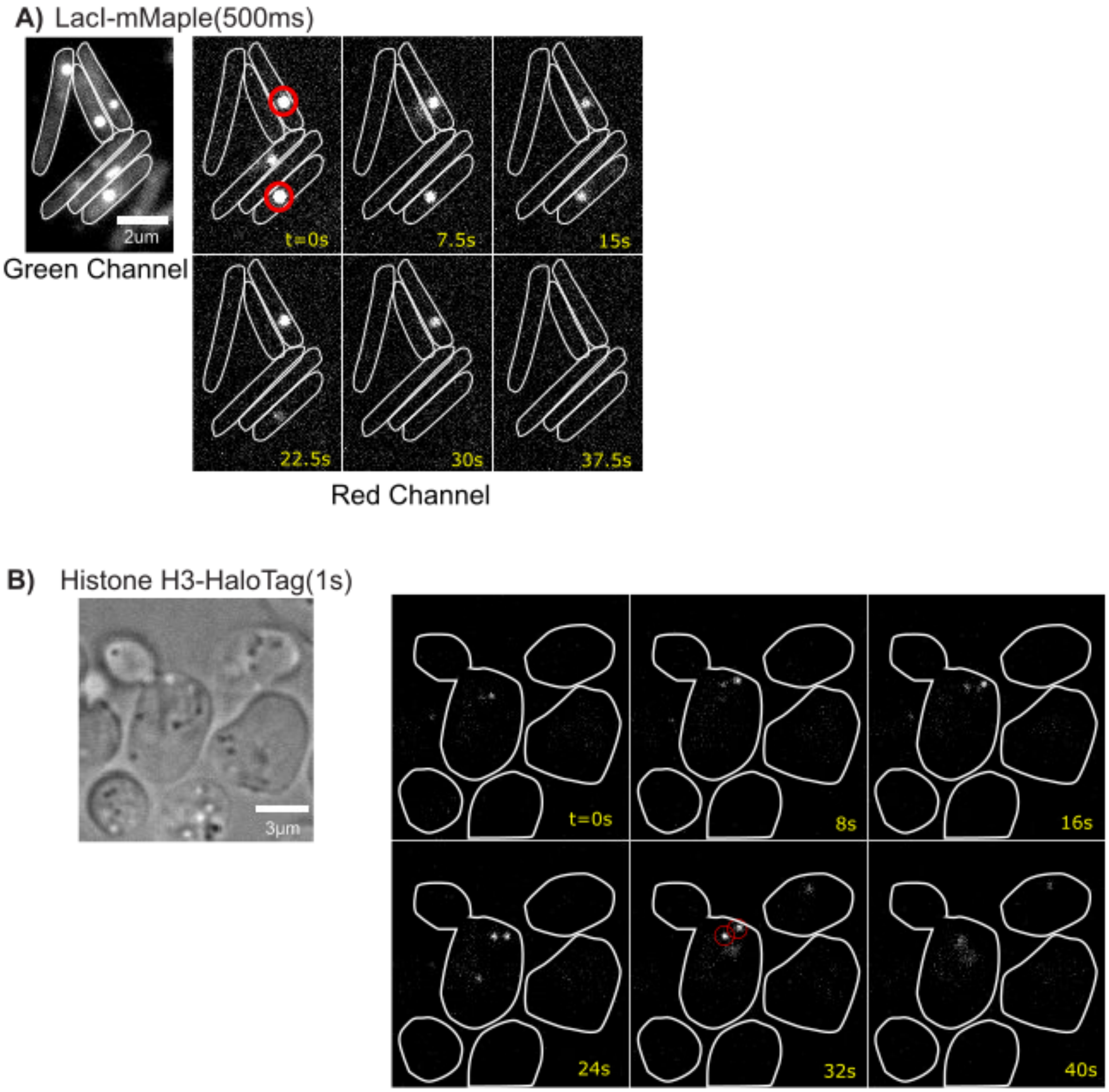

Figure S2. A) Example of timelapse for LacI-mMaple collected with continuous exposure acquisition. B) Example of timelapse for Histone H3-HaloTag collected with 500ms exposure, and 1s time interval acquisition. Red circles indicate molecules classified as being bound by Bound2Learn.

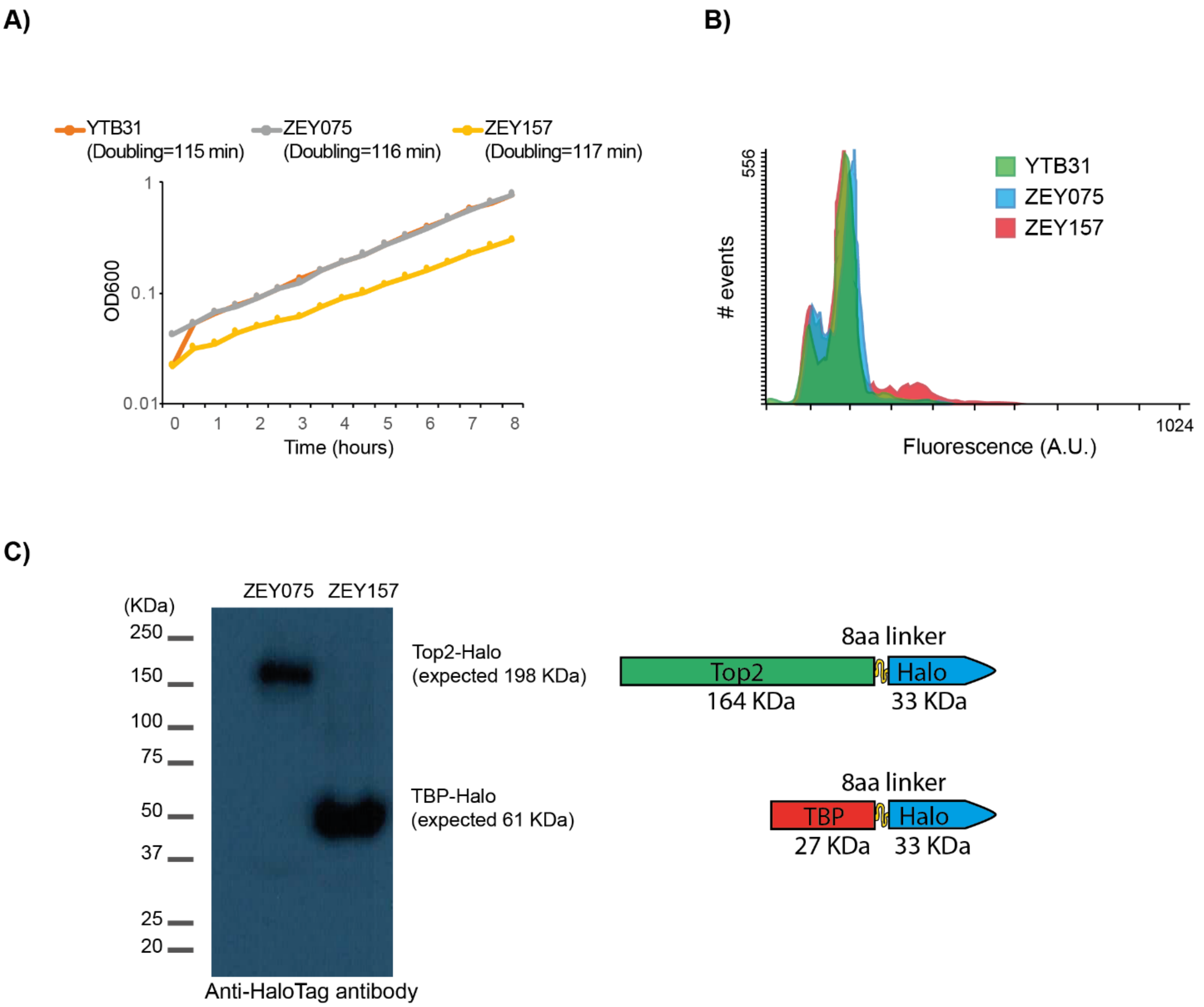

Figure S3. Characterization of Halo-tagged budding yeast strains. A) Strains YTB31, ZEY075 and ZEY157 were grown to exponential phase in SC, then diluted in fresh SC. OD600 measurements were taken every 30 min and used to plot growth curves. Doubling times were calculated from the slope of each curve. ZEY157 measurements were lower but the slope (and therefore doubling time) are very similar to the other strains. B) Exponential cultures of YTB31, ZEY075 and ZEY157 were grown to exponential phase, fixed in 70% ethanol then treated for flow cytometry. Samples were run on a FACSCalibur (Becton Dickinson) calibrated using parental haploid and diploid asynchronous exponential cultures. The proportion of cells in G1 and G2, as indicated by the two major peaks, are very similar across the 3 strains. C) Crude cell lysates from strains ZEY075 and ZEY157 were analyzed by SDS-PAGE and Western blot, using α-Halotag mouse monoclonal antibody (Promega) and a goat α-mouse HRP-conjugated secondary antibody (Promega). One single band is seen for each strain at a size close to that expected for the Halo-tagged fusion of each protein, with no detectable amount of free Halo (expected size 34 KDa). No bands at all were observed in YTB31, which does not have any Halo-tagged protein.

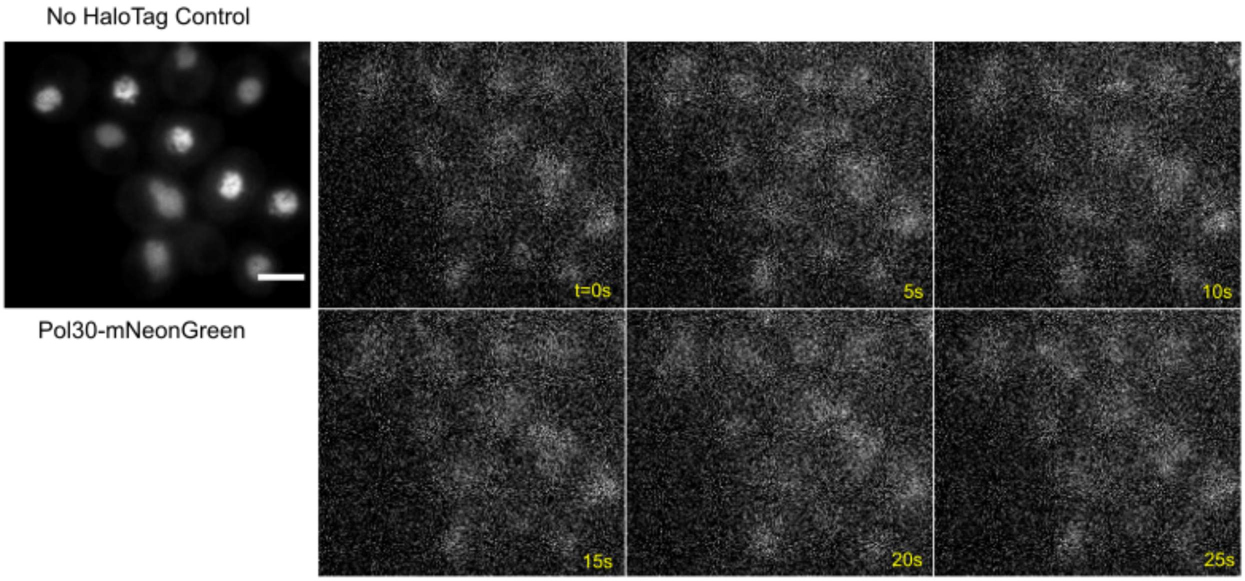

Figure S4. Example of timelapse for no HaloTag control (strain ZEY098), which had no HaloTagged protein but still had Pol30-mNeonGreen. Scale bar = 3um. Data collected with continuous exposure acquisition.

Training Data	Number of Trees	Minimum Leaf Size	Predictors to Sample at Node	Bag Fraction	OOB error	Number of Tracks
***E.coli* (simulation)**						
500ms (ML model 1)	6000	50	2	0.5	0.034	1263
500ms (ML model 2)	6000	50	2	0.5	0.0079	1263
100ms (ML model 1)	6000	50	2	0.5	0.0195	1075
100ms (ML model 2)	6000	50	2	0.5	0.014	1075
**Budding Yeast (simulation)**						
500ms (ML model 1)	10000	50	2	0.8	0.03	967
500ms (ML model 2)	10000	50	2	0.8	0.0269	967
**LacI**						
500ms (ML model 1)	10000	70	2	0.5	0.1634	1438
500ms (ML model 2)	10000	70	2	0.5	0.0709	1438
**Histone H3**						
500ms, 1s time interval (ML model 1)	6000	50	2	0.5	0.1151	1251
500ms, 1s time interval (ML model 2)	6000	50	2	0.5	0.036	1251

Table S1. Parameter values used to construct random forests.

**Training Data**	**Tbleach (s)**	**Tbound(s)**	**Tsearch(s)**	**D_{mobile} (um^2/s)**	**D_{bound} (um^2/s)**	**Mobile fraction**	**Bound Fraction**	**Integrated Spot Intensity**
**Ecoli**								
500ms exposure (no time interval)	10	100	100	0.5	0.005	0.3	0.7	3000
100ms exposure (no time interval)	2	100	100	0.5	0.005	0.3	0.7	3000
**Budding Yeast**								
500ms exposure (no time interval)	10	100	100	0.5	0.005	0.3	0.7	3000
**Experimental**								
**Ecoli**								
500ms exposure (1s time interval)	20	8	10000000	0.5	0.005	0.5	0.5	3000
500ms exposure (1s time interval, lower spot intensity)	20	8	10000000	0.5	0.005	0.5	0.5	2000
100ms exposure (no time interval)	2	1	10000000	0.5	0.005	0.7	0.3	3000
100ms exposure (no time interval, higher D_{mobile})	2	1	10000000	5	0.005	0.7	0.3	3000
100ms exposure (no time interval, mixed bound population)	10	1s/7s	10000000	0.5	0.005	0.1	0.45/0.45	3000
**Budding Yeast**								
500ms exposure (1s time interval)	20	8	10000000	0.5	0.005	0.5	0.5	3000
500ms exposure (1s time interval, lower spot intensity)	20	8	10000000	0.5	0.005	0.5	0.5	2000

Table S2 – Parameters used for simulations of training data and experimental data.

E.coli	Spot Intensity = 3000	Spot Intensity = 2000
**1s Interval (500ms exposure)**		
Bound Time [95% Confidence Interval]	6.76[5.41, 8.59]	7.21[5.65, 8.95]
Number of tracks predicted to be bound	169	159
Prediction Accuracy	0.93	0.99
Recovery Error	0.11	0.11
**100ms Exposure (no time interval)**		
Bound Time [95% Confidence Interval]	0.97[0.75, 1.21]	
Number of tracks predicted to be bound	156	
Prediction Accuracy	0.85	
Recovery Error	0.18	
**100ms Exposure (no time interval). Predicted using 500ms Training Data**		
Bound Time [95% Confidence Interval]	0.96[0.75, 1.22]	
Number of tracks predicted to be bound	158	
Prediction Accuracy	0.86	
Recovery Error	0.16	
**100ms Exposure (no time interval). D_{mobile} = 5um^2/s**		
Bound Time [95% Confidence Interval]	0.90[0.71, 1.13]	
Number of tracks predicted to be bound	142	
Prediction Accuracy	0.98	
Recovery Error	0.08	
**Diffusion Coefficient Analysis - 1s Interval (500ms exposure)**		
Bound Time [95% Confidence Interval]	3.72[3.05,4.46]	5.06 [3.97, 6.33]
Number of tracks predicted to be bound	291	203
Prediction Accuracy	0.54	0.79
Recovery Error	0.12	0.096
**Budding Yeast**		
Bound Time [95% Confidence Interval]	7.26[5.97, 8.77]	7.17[5.92, 8.82]
Number of tracks predicted to be bound	232	227
Prediction Accuracy	0.97	0.97
Recovery Error	0	0

Table S3. Results for simulated experimental data.

Protein	Time Interval	Mean Track Duration (s)	N
LacI	0.5	15.09 (13.42, 16.75)	316
LacI	0.5	10.91 (8.74, 13.64)	129
ε	1	7.26 (6.08, 8.84)	135
ε	1	7.74 (6.14, 9.96)	78
β	1	18.55 (16.14, 20.95)	229
β	1	15.19 (12.87, 19.52)	160
β	5	45.47 (40.36, 52.28)	279
β	5	48.51 (41.07, 56.72)	158
Histone H3	0.5	12.19 [10.71,13.87]	242
Histone H3	1	21.74 [18.41, 26.50]	123
TBP	0.5	6.18 [5.55,6.79]	495
TBP	0.5	5.32[4.61, 6.07]	234
Top2	1	11.49 [7.77, 19.05]	43
Top2	1	13.57[10.69, 17.62]	51

Table S4. Results with *E.coli* and budding yeast data. The 95% confidence are presented next to the estimates.

Strain	Genotype
**BY4741**	MATa his3Δ1 leu2Δ0 met15Δ0 ura3Δ0
**BY4742**	MATα his3Δ1 leu2Δ0 lys2Δ0 ura3Δ0
**BY4743**	MATa/α his3Δ1/his3Δ1 leu2Δ0/leu2Δ0 LYS2/lys2Δ0 met15Δ0/MET15 ura3Δ0/ura3Δ0
**YTB31**	MATα his3Δ1 leu2Δ0 lys2Δ0 ura3Δ0 POL30-mNeonGreen-Nat
**YTK1414**	MATa/α his3Δ1/his3Δ1 leu2Δ0/leu2Δ0 LYS2/lys2Δ0 met15Δ0/MET15 ura3Δ0/ura3Δ0 PDR5/pdr5Δ::KanMX
**ZEY098**	MATa his3Δ1 leu2Δ0 LYS2 met15Δ0 ura3Δ0 pdr5Δ0::KanMX POL30-mNeonGreen-Nat
**ZEY075**	MATa his3Δ1 leu2Δ0 LYS2 met15Δ0 ura3Δ0 pdr5Δ0::KanMX POL30-mNeonGreen-Nat TOP2-Halo-HygB
**ZEY157**	MATα his3Δ1 leu2Δ0 lys2Δ0 MET15 ura3Δ0 pdr5Δ0::KanMX POL30-mNeonGreen-Nat SPT15-Halo-HygB
**YTK1434**	MATa his3Δ1 leu2Δ0 met15Δ0 ura3Δ0 pdr5Δ0::KanMX HHT1-Halo-URA3

Table S5. Strains used**.** The *S. cerevisiae* strains used in this study are listed along with their genotypes.

Primer	Description	Sequence
**TB81**	C-terminal mNeonGreen tagging of PCNA (F)	cctacagtttttcttggctcctaaatttaatgacgaagaaGGTGACGGTGCTGGTTTA
**TB82**	C-terminal mNeonGreen tagging of PCNA (R)	tttattatttttagtatacaactatatagataatttacatCACAGGAAACAGCTATGACC
**TB98**	Screen C-terminal tag of PCNA (F)	AGAGTTGGTATCAGGCTCTC
**TB99**	Screen C-terminal tag of PCNA (R)	AAGCTGATATTTAACGCATCTTAG
**TOP2insF**	C-terminal Halo tagging of Top2 (F)	aggaaaaccaaggatcagatgtttcgttcaatgaagaggatGGTGACGGTGCTGGTTTA
**TOP2insR**	C-terminal Halo tagging of Top2 (R)	acatataaaaagaatggcgctttctctggataaatattatCACAGGAAACAGCTATGACC
**TOP2seqF**	Screen C-terminal tag of Top2 (F)	ACTATCTGGTGAAAGCGACC
**TOP2seqR**	Screen C-terminal tag of Top2 (R)	ACGATGTTTTTCGCCCAGGC
**NK46**	C-terminal Halo tagging of Spt15 (F)	tgaagctatataccctgtgctaagtgaatttagaaaaatgGGTGACGGTGCTGGTTTAAT
**NK47**	C-terminal Halo tagging of Spt15 (R)	aatagaaaaccttttttttcttttcgtctactccttccccaCAGTATAGCGACCAGCATTC
**NK50**	Screen C-terminal tag of Spt15 (F)	CTCCTATGAGCCAGAATTG
**NK51**	Screen C-terminal tag of Spt15 (R)	CTCCTATGAGCCAGAATTG

Table S6. Primers used in this study, with a short description and their sequence.
